# Hepatitis B Virus Cure: Targets and Future Therapies

**DOI:** 10.3390/ijms22010213

**Published:** 2020-12-28

**Authors:** Hye Won Lee, Jae Seung Lee, Sang Hoon Ahn

**Affiliations:** 1 Department of Internal Medicine, College of Medicine, Yonsei University, Seoul 03722, Korea; lorry-lee@yuhs.ac (H.W.L.); sikarue@yuhs.ac (J.S.L.); 2 Institute of Gastroenterology, College of Medicine, Yonsei University, Seoul 03722, Korea; 3 Yonsei Liver Center, Severance Hospital, Seoul 03722, Korea

**Keywords:** hepatitis B, treatment, cure, target

## Abstract

Chronic hepatitis B virus (HBV) infection is a major global health problem. It can cause progressive liver fibrosis leading to cirrhosis with end-stage liver disease, and a markedly increased risk of hepatocellular carcinoma. In the last two decades, substantial progress has been made in the treatment of chronic hepatitis, B. However, HBV is often reactivated after stopping nucloes(t)ide analogues because antivirals alone do not directly target covalently closed circular DNA, which is the template for all viral RNAs. Therefore, although currently available antiviral therapies achieve suppression of HBV replication in the majority of patients, hepatitis B surface antigen (HBsAg) loss and seroconversion is rarely achieved despite long-term antiviral treatment (HBsAg loss of less than 10% in 5 years). Various clinical trials of agents that interrupt the HBV life cycle in hepatocytes have been conducted. Potential treatment strategies and new agents are emerging as HBV cure. A combination of current and new anti-HBV agents may increase the rate of HBsAg seroclearance; thus, optimized regimens must be validated. Here, we review the newly investigated therapeutic compounds and the results of preclinical and/or clinical trials.

## 1. Introduction

Hepatitis B virus (HBV) infection is a leading cause of chronic liver disease worldwide. Antiviral agents, such as interferon and nucleos(t)ide analogues (NAs), have been used to treat patients with chronic hepatitis B (CHB). Antiviral treatment strongly suppresses HBV replication and slows progression to cirrhosis and hepatocellular carcinoma (HCC). However, current antivirals are not curative, because covalently closed circular DNA (cccDNA) persists in the hepatocyte nucleus [1]. Thus, new treatment targets against HBV are needed. There are approximately 30 candidates under investigation as new treatments of HBV infection [2].

HBV is a small, enveloped, and partially double-stranded DNA virus of the Hepadnaviridae family that infects hepatocytes, establishes its replication cycle, and persists in the nucleus [3]. The HBV virion is a lipid-based spherical structure on which three viral envelope proteins (small, middle, and large) are exposed [3]. The large envelope protein, which contains the receptor-binding domain, is involved in viral entry into cytoplasm by receptor-mediated endocytosis using the sodium taurocholate co-transporting polypeptide (NTCP) receptor in the hepatocyte membrane [4,5].

The viral envelope surrounds a nucleocapsid that contains a partially double-stranded, relaxed circular DNA (rcDNA) genome [6]. In the cytoplasm of HBV-infected hepatocytes, the nucleocapsid is transported through the nuclear pore complex by microtubule-mediated transport. Following capsid dissociation via unknown factors, rcDNA is released into the nucleus and is converted to cccDNA by host factors, forming a stable mini-chromosome [7,8]. The cccDNA is transcribed into pregenomic RNA (pgRNA), which is translated to the nucleocapsid protein and the HBV DNA polymerase and serves thereafter as the template for rcDNA synthesis. The nucleocapsid with partially double-stranded HBV DNA is enveloped and the virion is secreted. A part of the nucleocapsid is recycled into the nucleus and the rcDNA is converted to cccDNA. Each step of the HBV lifecycle is a potential therapeutic target for inhibiting HBV replication and reducing HBV infectivity (e.g., perinatal infection). 

## 2. What is the Definition of an HBV Cure?

Substantial progress has been made in the treatment of CHB in the last two decades. There are currently nine approved drugs for the treatment of CHB, including two formulations of interferon (IFN)—conventional and pegylated IFN (PegIFN)—and seven NAs: lamivudine, telbivudine, adefovir, entecavir, tenofovir disoproxil fumarate (TDF), tenofovir alafenamide fumarate (TAF), and besifovir dipivoxil (only in Korea).

The primary treatment goals for CHB are to prevent disease progression and increase survival. The therapeutic goals of current antiviral treatment are mainly virologic and biochemical responses related to the improvement of clinical outcomes [9]. A virologic response during NA therapy is defined as undetectable HBV DNA based on assays with a lower limit of detection of 10–20 IU/mL in blood. With IFN-based treatment, a virologic response is defined as a serum HBV DNA level of less than 2000 IU/mL when assessed at 6 months after the start of treatment and at the end of therapy. A biochemical response is defined as normalization of serum alanine aminotransferase. A normalization of alanine aminotransferase with a reduction of HBV viral load is an important goal to be achieved. However, these treatments do not generally promise a functional cure of CHB, which is an ideal goal of antiviral treatment.

A functional cure, defined as a sustained hepatitis B surface antigen (HBsAg) loss or seroconversion based on assays with a lower limit of HBsAg detection of 0.05 IU/mL, is a rare event in the natural history of CHB that is associated with a reduced risk of HCC [10,11]. In a population-based cohort study, the cumulative HCC incidence rate was significantly lower in participants with HBsAg seroclearance than in HBsAg-persistent carriers [12]. The HCC risk was similarly low after either spontaneous or NA-induced HBsAg seroclearance [13]. Complete cure is defined as an elimination of cccDNA together with durable HBsAg loss and undetectable serum HBV DNA. Although liver biopsy is needed to measure intrahepatic cccDNA activity, serum biomarkers that reflect cccDNA levels are needed instead. As candidates, HBV RNA, hepatitis B core-related antigen, and/or quantitative HBsAg have been investigated [14]. Quantification and ratio of large and middle proteins of HBsAg also showed specific patterns across different phases of hepatitis B that would predict the viral activity [15].

## 3. Targets for an HBV Cure

**Figure 1:**
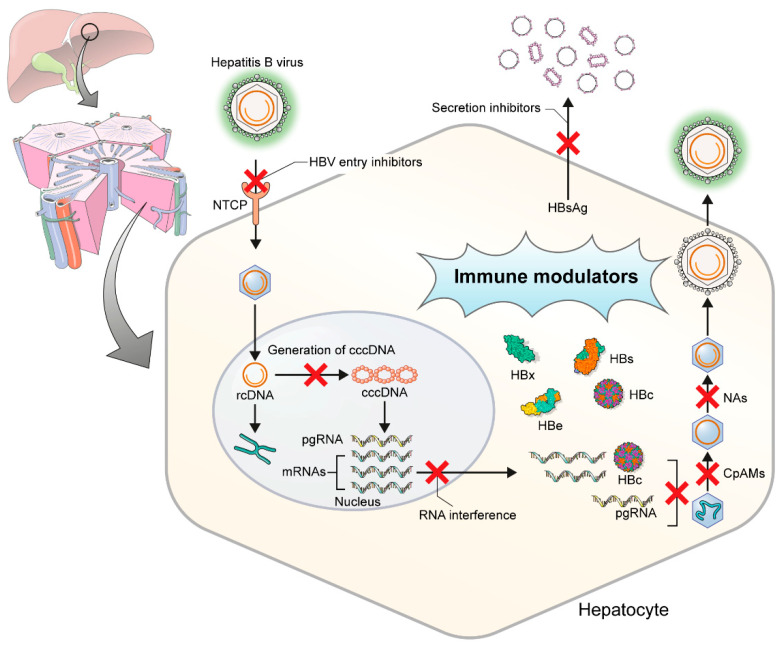
Targets of hepatitis B virus replication in hepatocytes. HBV, hepatitis B virus; NTCP, Na(sodium) taurocholate co-transporting polypeptide; rcDNA, relaxed circular DNA; cccDNA, covalently closed circular DNA; pgRNA, pregenomic RNA; CpAMs, core protein assembly modulators; NAs, nucleos(t)ide analogues.

Several drugs are now under development that directly target the HBV replication cycle or enhance the human immune response (Figure 1). New drugs for HBV cure include agents that directly target virus life cycle or those that indirectly modulate host factor/host immune response (Table 1). 

### 3.1. Direct-Acting Antivirals 

#### 3.1.1. Entry Inhibitors

Bulevirtide (formerly Myrcludex B) is a synthetic N-acetylated lipopeptide that contains the NTCP-binding pre-S1 domain of the large HBsAg envelope protein. A nanomolar range of affinity makes bulevirtide competitively bind to NTCP and block de novo HBV infection by competing with viral pre-S1 motif to bind with NTCP [16,17]. Bulevirtide was recently approved in the European Union for the treatment of chronic hepatitis delta virus (HDV) in HDV RNA-positive patients [18]. In a phase IIa study, 40 hepatitis B e antigen (HBeAg)-negative patients were randomized to receive bulevirtide at one of five dosages (0.5, 1, 2, or 5 mg for 12 weeks, and 10 mg once daily for 24 weeks). Bulevirtide was administered by subcutaneous injection. A decline in HBV greater than 1 log was observed in six out of eight patients in the 10 mg dosing group and in seven out of twenty-one patients in the lower dosing groups. However, no patient achieved HBsAg loss [19]. To date, most studies of bulevirtide have focused on chronic HDV infection. Sixty patients with HBV/HDV coinfection were randomized to receive PegIFN alone, PegIFN plus bulevirtide 2 or 5 mg, or bulevirtide 2 mg for 48 weeks [20]. Among the 60 patients, 20% of patients in the PegIFN plus bulevirtide 2 mg group achieved HBsAg loss at 24 weeks off therapy, and 27% achieved HBsAg loss at 48 weeks off therapy. In recent data (NCT03546621), 30 patients were randomized to PegIFN plus bulevirtide 10 mg once per day for 48 weeks, or TDF plus bulevirtide 5 mg twice a day for 48 weeks, together with sequential TDF for up to 72 weeks. At week 72, a higher proportion of the bulevirtide 5 mg plus TDF group showed undetectable HDV RNA, but HBsAg was undetectable in a lower proportion. The two groups showed the same proportions of alanine aminotransferase (ALT) normalization. There were no serious treatment-related adverse events or discontinuation of treatment. Most of these studies have been applied to HBV/HDV coinfection, but we expect a potent synergistic effect on patients with CHB when entry inhibitors and other antivirals are combined.

#### 3.1.2. Core Protein Allosteric Modulators

HBV core protein is essential for HBV pgRNA packaging and reverse transcription. Several compounds referred to as core protein allosteric modulators (CpAMs, also known as capsid assembly modulators in the literature) are under investigation. Depending on the chemical structure of CpAMs, unstable aberrant capsids or empty capsids can be formed. Two classes of CpAMs have been identified based on their mechanism of action [21]. Class I CpAMs, typified by heteroaryl dihyropyridines, increase the kinetics of capsid formation and lead to the formation of misassembled capsids. Class II CpAMs are typified by phenylpropenamides, which accelerate capsid assembly and form morphologically normal capsids that are empty and lack viral pgRNA and HBV polymerase.

NVR 3–778 was the first-in-class CpAM evaluated either alone or in combination with PegIFN. In a phase I study of HBeAg-positive patients with CHB without cirrhosis, reductions in both HBV DNA and HBV RNA were greater in the group with NVR 3–778 plus PegIFN than in the group with NVR 3–778 or PegIFN alone [22]. However, viral rebound was observed after drug cessation.

JNJ-6379 (JNJ-56136379) is a class II CpAM that binds to the HBV core protein and disrupts early- and late-stage processes in the HBV life cycle [14]. In a phase I study of treatment-naïve patients with CHB, a 4-week administration of JNJ-6379 was tolerated, showed dose-dependent pharmacokinetics, and had potent antiviral activity (decreases in HBV DNA and HBV RNA) [23]. Primarily, JNJ-6379 interferes with capsid assembly kinetics, preventing encapsidation of pgRNA and blocking HBV replication (late step in the viral life cycle). In addition, JNJ-6379 can also inhibit de novo formation of cccDNA, potentially interfering with the capsid disassembly process. JNJ-6379 blocks production of Dane particles and RNA-containing particles. However, since it does not target HBsAg, subviral particles of HBsAg can still be released to the blood. A phase II study is ongoing.

RO7049389 is an oral small molecule and class I HBV CpAM that induces formation of abnormal HBV core aggregates. In a recent phase I study, RO7049389 was administered to HBV patients at different dose regimens for 28 days and resulted in a robust decline in median HBV DNA (2.7 to 3.0 log_10_ IU/mL), which is promising for virus suppression. Viral rebound was observed after stopping treatment [24].

ABI-H0731 (vebicorvir) is a potent and selective class II CpAM. Thirty-eight non-cirrhotic patients were randomly assigned to receive ABI-H0731 or placebo and treated once daily for up to 28 days in a double-blind, placebo-controlled study. In the second study, vebicorvir 300 mg plus entecavir or entecavir plus placebo daily was given to treatment-naïve HBeAg-positive patients [25]. Compared to entecavir plus placebo, the combination of vebicorvir plus entecavir resulted in significant declines at week 12 in both HBV DNA and HBV RNA. In recent data (NCT03576066), 100% of HBeAg-negative CHB patients (n = 18) had virologically suppressed HBV DNA plus pgRNA levels <20 IU/mL after 48 weeks of treatment with vebicorvir 300 mg once daily in combination with oral entecavir, TDF or TAF. Based on the possibilities of using capsid inhibitors plus NAs to substantially decrease HBV DNA levels, a phase III study is in preparation. Long-term studies are needed to determine whether CpAMs can eliminate HBsAg, HBeAg and/or cccDNA.

#### 3.1.3. RNA Interference

RNA interference (RNAi) can target HBV transcripts directly and induce their degradation. RNAi is a highly specific and efficient method of post-transcriptional gene silencing [26]. The synthetic small interfering RNA (siRNA) interferes with the expression of a specific target gene by degrading mRNA. Unlike NA therapy, siRNA shuts down HBsAg production and may lead to the restoration of the immune response through rapid HBsAg reduction and breakdown of immune tolerance. A major limitation of this drug is the issue of delivery, and the lack of a reduction in cccDNA. Antisense oligonucleotides are small nucleic acids that are complementary to the target transcript and act through steric hindrance and/or RNA degradation by ribonuclease H cleavage. 

ARC-520 was investigated in two randomized, multicenter studies of NA-experienced patients who were HBeAg-negative or HBeAg-positive [27]. HBsAg levels were significantly reduced in the high-dose groups (2 mg/kg ARC-520 combined with NAs) compared to a placebo group, with mean reductions of 0.38 log IU/mL in HBeAg-negative patients and 0.54 log IU/mL in HBeAg-positive patients; the reductions persisted for approximately 85 days and >85 days after the last dose. A modified RNAi, JNJ-3989 (formerly ARO-HBV), contains two RNAi that are both conjugated to N-acetyl galactosamine to facilitate uptake by the liver. ARO-HBV induced rapid declines in HBV DNA and HBsAg in patients with CHB [28]. In recent data, JNJ-3989 injections with entecavir or TDF resulted in maintained HBsAg declines ≥1 log_10_ IU/mL in 39% (15/38) of patients with CHB.

An alternative approach to blocking viral protein expression is to use liver-directed antisense oligonucleotides. IONIS-HBVRx (GSK3228836) and IONIS-HBVLRx (GSK33389404) allow delivery of antisense molecules to the liver via the hepatocyte-expressed asialoglycoprotein. This approach may reduce off-target toxicities associated with antisense oligonucleotides [29]. 

#### 3.1.4. Inhibition of HBsAg Release

The most abundant viral antigen in the blood is HBsAg, which plays an important role in preventing immune control of HBV [8,30]. Circulating HBsAg is almost entirely in the form of non-infectious HBV subviral particles (SVPs) [31,32,33]. Because these particles are produced independently of viral replication, the viral antigen is difficult to target using the therapies approved to date.

Nucleic acid polymers, such as REP 2139, block the release of HBsAg from infective hepatocytes by selectively targeting the assembly and/or secretion of SVPs [34]. REP 2139 naturally enters liver cells (hepatocytes), where it prevents the assembly of SVPs in any hepatocyte producing these particles. This mechanism effectively hinders the replenishment of HBsAg in the blood and reduces HBsAg levels within hepatocytes. The overall antiviral effect of REP 2139 allows the body to clear HBsAg and thereby reduce or remove the inhibition of immune control caused by this viral antigen. The current formulation of REP 2139 (REP 2139-Mg) induces few to no side effects and is typically administered once every week for 48 weeks by intravenous infusion in combination with other antiviral agents. Moreover, REP 2139-Mg is expected to be equally effective with a once-weekly injection under the skin (subcutaneous injection), a regimen that will be used in future trials.

Recently, the safety and efficacy of the addition of either REP 2139-Mg or REP 2165-Mg to a backbone of TDF and PegIFN was investigated in an open-label, randomized phase II study of HBeAg-negative CHB patients [35]. After the 48-week treatment, HBsAg levels were ≤0.05 IU/mL in 60% (24/40) of the patients. During a further 48-week of treatment-free follow-up, virologic control persisted in 32.5% (13/40) of patients. Among patients with persistent HBsAg seroconversion, functional cure was maintained in 35% (14/40). Thus, the administration of HBsAg release inhibitors together with immune modulators may be an effective combination regimen.

#### 3.1.5. Neutralization

Hepabig gene (lenvervimab) is a recombinant human monoclonal antibody that binds to HBsAg to neutralize HBV. This compound not only inhibits viral entry into hepatocytes but also enhances the immune response by significantly reducing HBsAg levels. The mechanisms involve the neutralization of circulating virion or surface antigen by the formation of immune complexes and the inhibition of viral re-entry by binding to HBsAg. In a prospective, open-label phase I trial, single and multiple doses of lenvervimab were administered in four different doses (80,000 IU, 120,000 IU, 180,000 IU or 240,000 IU) to HBeAg-positive patients, and virological suppression occurred in HBeAg-negative CHB patients naturally or with NA therapy [36]. Lenvervimab showed good tolerability as well as a correlation between the baseline HBsAg level and sustained HBsAg loss. It is now undergoing a double-blind, randomized, phase IIa study to evaluate its efficacy and safety when administered in combination with NAs. 

#### 3.1.6. Inhibitors of cccDNA

cccDNA serves as the template for viral transcription, and pgRNA as the template for viral replication. Thus, disabling cccDNA can be an effective curative option for HBV infection. Numerous small molecules have been developed as sequence-specific RNA-guided nucleases and proteins that can putatively block the formation, enhance the destruction, and silence the transcription of cccDNA while stimulating cell division [37]. These include a cleaving sequence-specific DNA targets using the transcription activator-like (TAL) effector nucleases (TALENs) or a gene-editing using the clustered regularly interspaced short palindromic repeats (CRISPR)/CRISPR-associated 9 (Cas9) system, showing antiviral efficacy [38,39]. Epigenetic modification by histone modification and cccDNA methylation can modify actively transcribed DNA to an inactive status without changing the DNA itself [40]. Potential inhibitors of cccDNA such as directly gene-editors, epigenetic modifiers and DNA destabilizer are on development, however, they have yet to be evaluated in clinical trials.

### 3.2. Immune Modulatory Therapies or Indirect Antivirals

A weak innate and HBV-specific immunologic response occurs in patients with CHB [21]. Chronic HBV infection leads to T-cell dysfunction (“exhaustion”), a state characterized by poor effector cytotoxic activity, impaired cytokine production, and the sustained expression of multiple inhibitory receptors [41]. Thus, the identification of immunomodulatory targets is important in therapeutic strategies aimed at restoring HBV-specific immune responses with immunomodulatory agents. However, the results achieved thus far with immune modulators have been disappointing. 

#### 3.2.1. Toll-Like Receptor Agonists

Toll-like receptors (TLRs) are the initial sensors of viral infection and initiate the intracellular pathways that induce the production of antiviral mediators [42]. The activation of TLR-mediated pathways results in suppression of HBV replication and restoration of HBV-specific adaptive immunity. In a double-blind, randomized, placebo-controlled phase II study, patients received once-weekly oral vesatolimod (GS-0620, TLR-7 agonist) or placebo [43]. Vesatolimod induced an HBV-specific immune response, but without a significant decline in HBsAg. Selgantolimod (GS-9688, TLR-8 agonist) is now in a phase II study. Selgantolimod (1.5 mg or 3 mg once a day) was administered with NA therapy for 24 weeks, followed by NA therapy alone for 24 weeks. HBsAg loss was achieved in 5% (2/39) of the patients, and in 16% (3/19) of patients who were HBeAg-positive. Another study (NCT03615066) that tested selgantolimod plus TAF against a placebo plus TAF in viremic patients with CHB showed that selgantolimod is safe and well tolerated, with a decline in HBsAg levels to ≥0.3 log_10_ IU/mL in the selgantolimod plus TAF group. 

#### 3.2.2. Engineered T Cells

The adoptive transfer of newly engineered HBV-specific T cells may be a novel strategy [44]. Genetic reprogramming to create functional T cells to eliminate HBV-infected hepatocytes could be achieved through T-cell receptor (TCR) gene transfer or through the use of chimeric antigen receptor (CAR) T cells [21]. Tests of HBV-specific T cells with CAR or classic TCRs both in vitro and in HBV transgenic mice have shown selective elimination of HBV-infected cell lines and control of HBV replication with only transient liver damage, respectively [45,46]. 

#### 3.2.3. Immune Checkpoint Inhibitors

The efficacy of targeting checkpoint inhibitors, such as programmed cell death protein 1 (PD-1) and programmed death ligand 1 (PD-L1), has been demonstrated by the restoration of vigorous immune responses in patients with malignancies. However, there are concerns about the induction of autoimmunity or hepatitis flare via nonspecific activation of the immune system.

Anti-PD-L1 may be a therapeutic candidate in patients with CHB, as it is expected to restore antiviral T-cell functions [47]. In vivo blockade of the PD-1/PD-L1 pathway in CD8 T cells, in combination with entecavir treatment and DNA vaccination, was shown to enhance the function of virus-specific T cells [48]. A phase I pilot study evaluated anti-PD-1 (nivolumab) treatment with or without GS-4774 (therapeutic vaccine) in HBeAg-negative CHB patients. At week 24, 14% (3/22) of the patients had > 0.5 log_10_ reduction in HBsAg levels [49]. 

#### 3.2.4. Therapeutic Vaccine

The aim of therapeutic vaccines is to stimulate the host immune response to restore HBV-specific immune control while suppressing HBV replication and ultimately inducing HBsAg loss. However, these vaccines have been largely unsuccessful [50]. The only finding of efficacy was a reduction in HBV DNA levels at the end of follow-up in patients given a therapeutic vaccine plus the standard of care compared to those who received the standard of care alone. In contrast to the failure of previous vaccines that only target HBsAg, the development of newer DNA vaccines is attempting various prime-boost approaches.

Other DNA vaccines are currently under development, including heterologous prime-boost approaches, vaccines against multiple HBV proteins, and novel adjuvants [51]. INO-1800 is a vaccine consisting of a mixture of recombinant DNA encoding the HBsAg and the consensus sequence of the hepatitis B core/capsid protein antigen. INO-1800, alone or in combination with interleukin-12, is currently being investigated in patients with CHB (NCT02431312). Another candidate is JNJ-64300535, which is being tested in combination with NAs. Vaccines such as GS-4774 and TG-1050 are based on approaches using multiple HBV proteins, but they have yielded disappointing results in clinical trials. Nonetheless, preliminary data imply that therapeutic vaccines will be effective when administered in a combination approach. 

## 4. Future Perspectives

We expect that combination of new drugs may have a higher chance of inducing functional cure of HBV infection. Combination of current and new anti-HBV agents may increase rates of HBsAg seroclearance, but optimized regimens must be validated. A significant decrease in HBsAg levels was achieved with an RNA interference-based triple-combination therapy (JNJ-3989, JNJ-6379, and NAs) after 12 weeks of treatment [52]. Various therapeutic combinations can be considered: NAs plus one or two other agents, such as a CpAM, siRNA, HBV entry inhibitor, or cccDNA inhibitor in combination with a direct-acting antiviral agent; or an antiviral/inhibitor of viral antigen burden with an immunomodulatory or therapeutic vaccine. 

## 5. Summary

Several novel compounds are under development as cures for HBV infection, including direct-acting antiviral agents that are now in phase II clinical trials, which are HBV entry inhibitors, CpAMs, siRNA, HBsAg-release inhibitors or neutralizers, and inhibitors of cccDNA with different mechanisms of action. However, CHB is characterized by a weak innate immunity and HBV-specific immune response. To restore immunity, TLR agonists, engineered T cells, immune checkpoint inhibitors, and therapeutic vaccines can be considered. New immune modulators may have fewer side effects than interferon, but more data demonstrating their clinical benefits in HBsAg seroclearance are needed. A better chance of functional cure of HBV infection may come from a combination of new drugs that act via different mechanisms. Combinations that target the viral life cycle directly and induce host immunity are likely to be the most effective. Further studies are needed to demonstrate the safety and efficacy of these drugs. Global efforts for the functional cure of HBV infection hold promise.

## Figures and Tables

**Table 1:** Summary of new antiviral drugs for hepatitis B cure.

Target	Phase	Drug Name	Mode of Action	Company	Administration	NCT Number
Entry inhibitors	II	Bulevirtide	HBV entry inhibition	Hepateral Ltd	SC	NCT03852433
Core protein allosteric modulators	I	NVR 3–778	Assembly modulator	Novira Therapeutics, Inc.	Oral	NCT02401737
	II	GLS4	Core protein binding	Sunshine Lake Pharma Co., Ltd.	Oral	NCT04147208
	I	RO7049389	Core protein binding	Hoffmann-La Roche	Oral	NCT02952924
	II	JNJ-56136379 (JNJ-6379)	Assembly modulator	Janssen, Scotland	Oral	NCT03982186
	II	ABI-H0731 (Vebicorvir)	Core protein binding	Assembly Biosciences	Oral	NCT03780543
	II	ABI-H2158	Core protein binding	Assembly Biosciences	Oral	NCT04398134
	1	JNJ-64530440 (JNJ-0440)	Assembly modulator	Alios Biopharma Inc.	Oral	NCT03439488
	II	QL-007	Assembly modulator	Qilu Pharmaceutical Co., Ltd.	Oral	NCT04157699
RNA interference	II	ARC-520	RNA interference	Arrowhead Pharmaceuticals	IV	NCT02577029
	II	INOIS-HBVRx (GSK3228836)	Antisense oligonucleotides	GlaxoSmithKline	SC	NCT02981602
	Preclinical	INOIS-HBVLRx (GSK33389404)	Antisense oligonucleotides	GlaxoSmithKline	-	-
	II	VIR-2218	RNA interference	Vir Biotechnology, Inc.	SC	NCT04412863
	II	ARO-HBV (JNJ-3989)	RNA interference	Arrowhead Pharmaceuticals	SC	NCT03365947
	I	DCR-HBVS	RNA interference	Dicerna Pharmaceuticals, Inc.	SC	NCT03772249
Inhibition of HBsAg release	II	REP 2139-Ca	Inhibition of HBsAg release	Replicor Inc.	IV	NCT02726789
	II	REP 2139-Mg	Inhibition of HBsAg release	Replicor Inc.	IV	NCT02565719
HBsAg neutralization	II	GC 1102 (Lenvervimab)	Neutralization and inhibiting reentry	Green Cross Corporation	IV	NCT03801798
Inhibitors of cccDNA	Preclinical	TALENs	cccDNA disruption	-	-	-
	Preclinical	CRISPR-Cas9	cccDNA disruption	-	-	-
Toll-like receptor agonists	II	GS-9620 (vesatolimod)	TLR7 agonist	Gilead Sciences	Oral	NCT02166047
	II	GS-9688 (selgantolimod)	TLR8 agonist	Gilead, USA	Oral	NCT03615066
	I	RO7020531	TLR7 agonist	Hoffmann-La Roche	Oral	NCT02956850
Immune checkpoint inhibitors	I	Nivolumab	Anti-PD-1	PharmaEssentia	IV	NCT04638439
	I/II	REGN2810 (cemiplimab)	Anti-PD-1	Regeneron Pharmaceuticals	IV	NCT04046107
Therapeutic vaccines	I	INO-1800	DNA plasmids	Inovio Pharmaceuticals	E-IM	NCT02431312
	I	TG1050	HBV proteins	Transgene	SC	NCT02428400
	I	ChAdOx1 HBV	Adjuvanted ChAd and MVA vectored	Vaccitech Limited	IM	NCT04297917
	I	JNJ-64300535	DNA vaccines	Janssen Sciences Ireland UC	E-IM	NCT03463369
	II	GS-4774	DNA vaccines	Gilead Sciences	SC	NCT02174276

Abbreviations: HBV, hepatitis B virus; SC, subcutaneous; IV, intravenous; HBsAg, hepatitis B surface antigen; cccDNA, covalently closed circular DNA; TALENs, transcription activator-like effector nuclease; TLR, toll-like receptor; Anti-PD-1, anti-programmed cell death protein 1; IM, intramuscular; E-IM electroporation-mediated intramuscular. References: https://www.clinicaltrials.gov (updated on 20 December 2020).

## Data Availability

Not applicable.
